# Supplementary figures and images for: Distinct actin microfilament localization during early cell plate formation through deep learning-based image restoration
Source: Plant Cell Rep. 2025 May 8;44(6):115. doi: 10.1007/s00299-025-03498-7 (PMC12058911; doi:10.1007/s00299-025-03498-7)

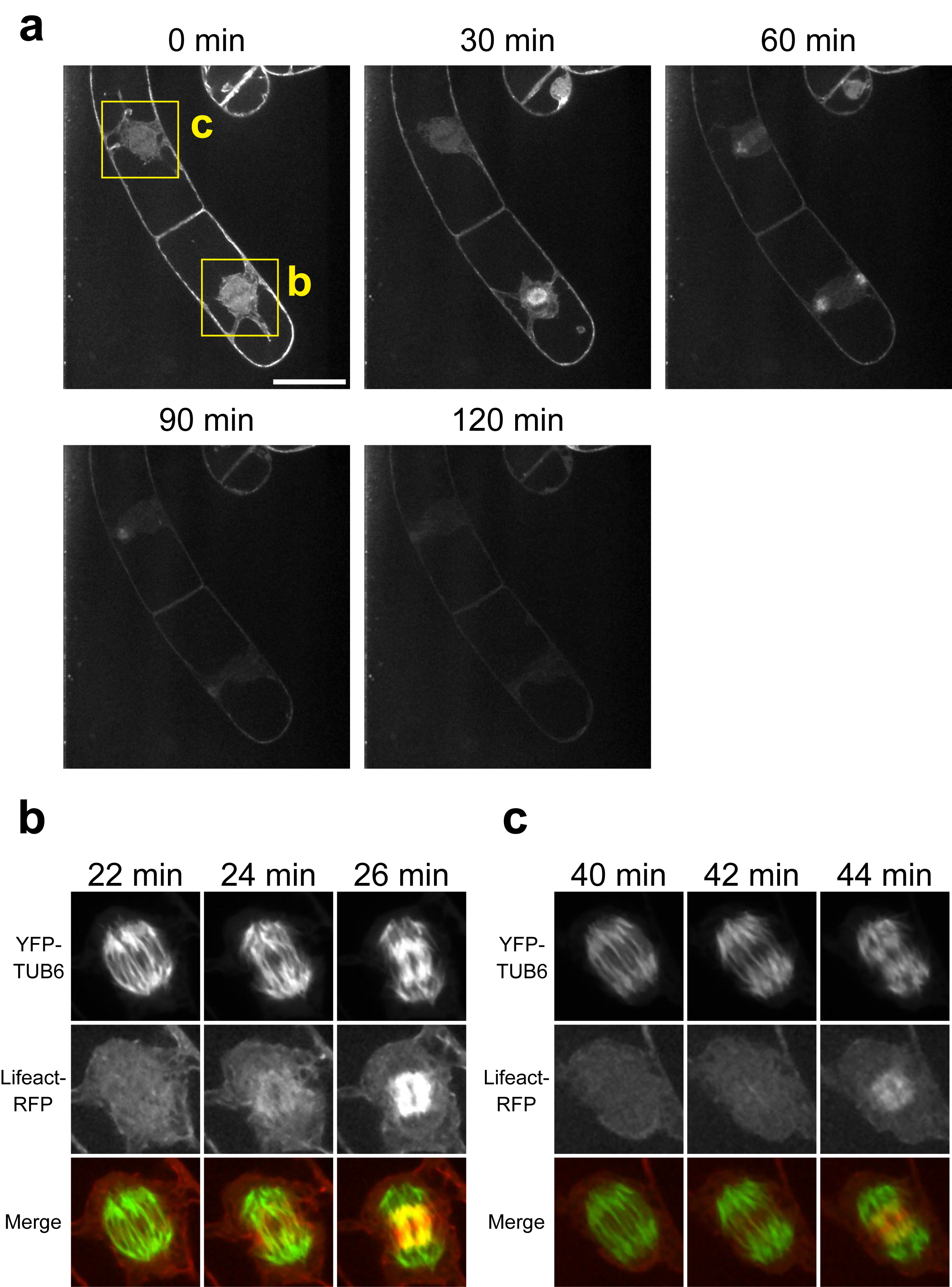

Supplement: Supplementary file 1 — Supplementary file1 Supplementary Fig. S1 Photobleaching during live-cell fluorescence imaging. (a) Time-lapse images of tobacco BY-2 cells with fluorescently labeled microtubules (YFP-TUB6) and actin microfilaments (Lifeact-RFP). (b) and (c) are enlarged images of the mitotic apparatus surrounded by the yellow squares in (a). The acquisition time interval was 2 min, and 40 optical sections (Z-interval of 1 µm) were obtained at each time point. Each time-lapse image is shown with a representative single optical section through the midplane of the division apparatus of four cells. The time point of the start of acquisition is indicated as t = 0. (b) Scale bar = 40 (TIF 15809 KB) [file 299_2025_3498_MOESM1_ESM.tif]

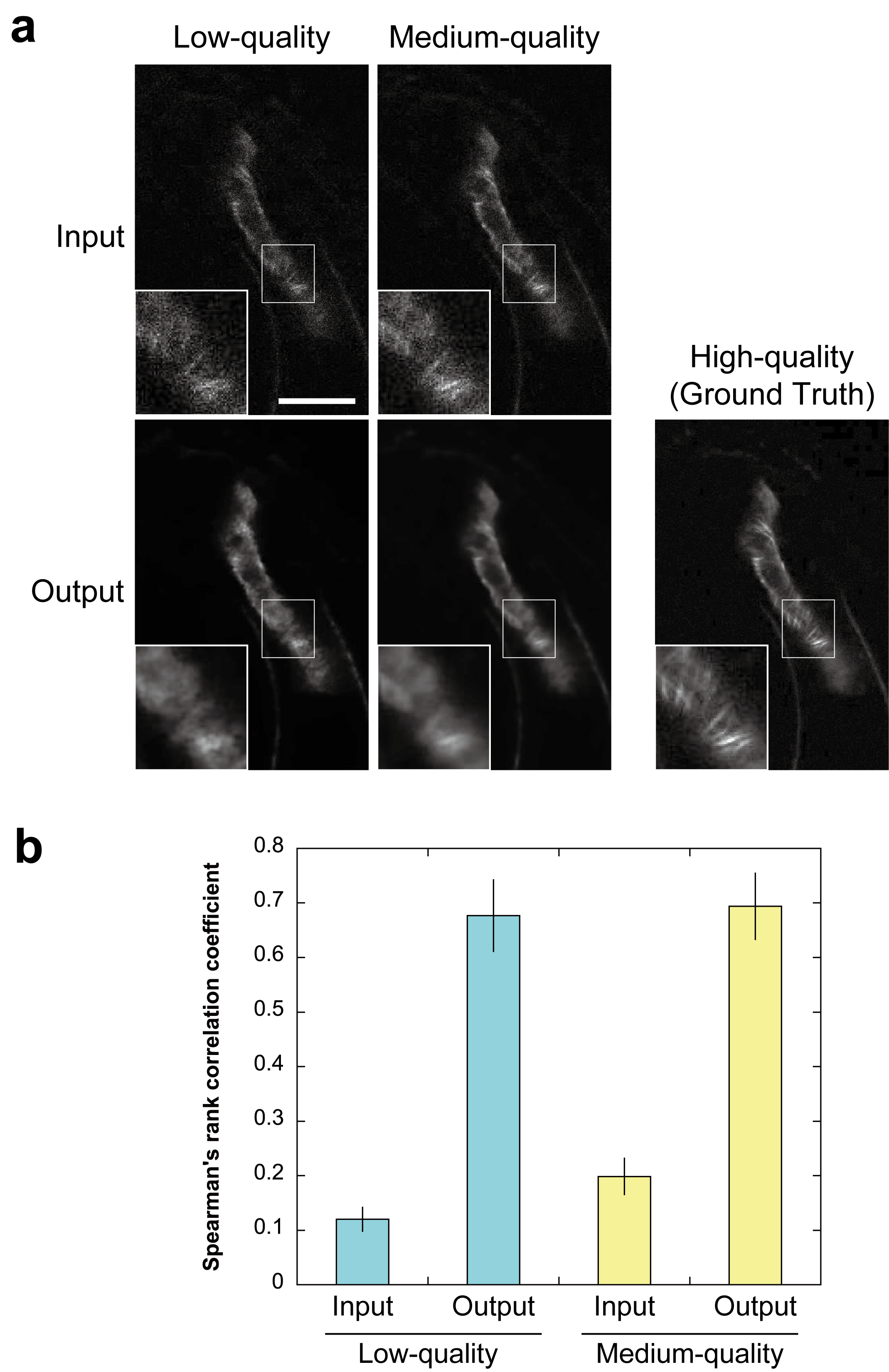

Supplement: Supplementary file 2 — Supplementary file2 Supplementary Fig. S2 Application of image restoration to the imaging of A. thaliana zygotes. (a) Representative images of A. thaliana zygote with fluorescently labeled microtubules (Clover-TUA6) before and after image quality restoration. Results for low- (left) and medium-quality (center) images are shown with “Input” (upper) and “Output” (lower) images. The corresponding high-quality image is shown in the lower right. The inset is an enlarged view of the white frames. (b) Results of the quantitative evaluation of image restoration accuracy in which “Input” and “Output” images were compared with the ground truth image, and their similarity was quantified using Spearman's rank correlation coefficient (SRCC). Error bars indicate standard deviation (n = 23 for each bar). Scale bar = 20 µm (TIF 6960 KB) [file 299_2025_3498_MOESM2_ESM.tif]
